# *Annals of general psychiatry* reviewer acknowledgement 2014

**DOI:** 10.1186/s12991-015-0044-4

**Published:** 2015-02-19

**Authors:** Konstantinos N Fountoulakis

**Affiliations:** Department of Psychiatry, School of Medicine, Aristotle University of Thessaloniki, Thessaloniki, Pylaia 55535 Greece

## Abstract

The editors of *Annals of General Psychiatry* would like to thank all of our reviewers who have contributed to the journal in volume 13 (2014).

**Monica Aas**

Norway

**Ranil Abeyasinghe**

Sri Lanka

**Ramesh Adhikari**

Nepal

**Jorge Almeida**

United States of America

**Keith Anderson**

United States of America

**Ion Anghelescu**

Germany

**Marc Auriacombe**

France

**Alasdair Barr**

Canada

**Anna Bartak**

Netherlands

**Thomas Beblo**

Germany

**Thomas Becker**

Germany

**Stefan Bleich**

Germany

**Pierre Blier**

Canada

**Tony Bogdanoski**

Australia

**Pascal Bonaventure**

United States of America

**Cynthia Bossie**

United States of America

**Sevasti Bostantjopoulou**

Greece

**Demetria Cain**

United States of America

**Andreas Carlborg**

Sweden

**Han Chae**

South Korea

**Weichiao Chang**

Taiwan

**Martin Cheatle**

United States of America

**Asbjørg Solberg Christophersen**

Norway

**Roberto Cilia**

Italy

**Lawrence Cohen**

United States of America

**Leonard Crocombe**

Australia

**Domenico De Berardis**

Italy

**Filip De Fruyt**

Belgium

**Marc de Somer**

United States of America

**Doug Delahanty**

United States of America

**Dimos Dimellis**

Greece

**Athanassios Douzenis**

Greece

**Tsachi Ein-Dor**

Israel

**Tim Esbenshade**

United States of America

**Ariel Eytan**

Switzerland

**Andrea Fagiolini**

Italy

**Beatriz Fagundo**

Spain

**Cleusa Ferri**

United Kingdom

**Julian Ford**

United States of America

**Emmanuel Fort**

France

**Wolfgang Gaebel**

Germany

**Sturla Gjesdal**

Norway

**Lydia Gomez Perez**

Spain

**Fragiskos Gonidakis**

Greece

**Giuseppe Grosso**

Italy

**Charles Hall**

United States of America

**Magnus Haraldsson**

Iceland

**Egbert Hartstra**

Belgium

**Stephan Heres**

Germany

**Jameson Hirsch**

United States of America

**Lisa Horowitz**

United States of America

**Yu-Shu Huang**

Taiwan

**Thomas Hyphantis**

Greece

**Miro Jakovljevic**

Croatia

**Georg Juckel**

Germany

**Walter Kalu**

United States of America

**Vagelis Karavelas**

Greece

**Siegfried Kasper**

Austria

**Takahiro Kato**

Japan

**Anastasia Keivanidou**

Greece

**Vasilios Kimiskidis**

Greece

**Batool Kirmani**

United States of America

**Tsuyoshi Kondo**

Japan

**Nastasios Konstantinidis**

Austria

**Stefan Kropp**

Germany

**Maarit Laaksonen**

Finland

**Stefan Leucht**

Germany

**Ming Li**

United States of America

**Juan Jose López-Ibor**

Spain

**Francisco López-Muñoz**

Spain

**Karina Lovell**

United Kingdom

**Ru-Band Lu**

Taiwan

**Amy Luke**

United States of America

**Melinda Madlena**

Hungary

**Barrie Marchant**

United States of America

**Miguel Martinez-Gonzalez**

Spain

**Teresa Mashego**

South Africa

**Francesco Matrisciano**

Italy

**Luigi Mazzone**

Italy

**Erin McClure**

United States of America

**Christian Meyer**

Germany

**Allison Milner**

Australia

**Marcellino Monda**

Italy

**Ali Montazeri**

Iran

**Jill Morrison**

United Kingdom

**Driss Moussaoui**

Morocco

**Elizabeta Mukaetova-Ladinska**

United Kingdom

**Masaaki Murakami**

Japan

**Yu Nakamura**

Japan

**Michiko Nakazato**

Japan

**David Ndetei**

Kenya

**Shusuke Numata**

Japan

**Tarek Okasha**

Egypt

**Koichi Otani**

Japan

**Eugenia Oviedo-Joekes**

Canada

**Bjorn Owe-Larsson**

Sweden

**Maryland Pao**

United States of America

**Antoine Pelissolo**

France

**Orion Penner**

Italy

**Concepcion Perez**

Spain

**Giulio Perugi**

Italy

**Anna Plaza**

Spain

**Mark-Jan Ploegstra**

Netherlands

**Antonio Preti**

Italy

**Radovan Prikryl**

Czech Republic

**Janice Probst**

United States of America

**Rosemary Purcell**

Australia

**Siri Ranlund**

United Kingdom

**Valdo Ricca**

Italy

**Stephan Rüegg**

Switzerland

**Andriy V Samokhvalov**

Canada

**Luiz Miguel Santiago**

Portugal

**Vedat Sar**

Turkey

**Gianluca Serafini**

Italy

**Mitsue Shimizu**

Japan

**Jay Shore**

United States of America

**Iris Sommer**

Netherlands

**Eric Storch**

United States of America

**Beth Stuart**

United Kingdom

**David Taylor**

United Kingdom

**Takeshi Terao**

Japan

**Gretchen Tietjen**

United States of America

**Tineke van Veen**

Netherlands

**Joris Verster**

Netherlands

**Cheryl Vigen**

United States of America

**Jelena Vrublevska**

Latvia

**Florian Weck**

Germany

**Jacquie White**

United Kingdom

**Leenika Wijeratne**

Sri Lanka

**Thomas Wobrock**

Germany

**Paula Wye**

Australia

**Norio Yasui-Furukori**

Japan

